# Quasi-linear Cox proportional hazards model with cross- L_1_ penalty

**DOI:** 10.1186/s12874-020-01063-2

**Published:** 2020-07-06

**Authors:** Katsuhiro Omae, Shinto Eguchi

**Affiliations:** 1 Department of Clinical Biostatistics, Graduate School of Medicine, Kyoto University, Yoshida Konoe-cho, Kyoto, Japan; 2 The Institute of Statistical Mathematics, Midori-cho, Tachikawa, Tokyo Japan

**Keywords:** Cox’s proportional hazards model, Generalized average, Heterogeneity, Mixture model, Survival analysis

## Abstract

**Background:**

To accurately predict the response to treatment, we need a stable and effective risk score that can be calculated from patient characteristics. When we evaluate such risks from time-to-event data with right-censoring, Cox’s proportional hazards model is the most popular for estimating the linear risk score. However, the intrinsic heterogeneity of patients may prevent us from obtaining a valid score. It is therefore insufficient to consider the regression problem with a single linear predictor.

**Methods:**

we propose the model with a quasi-linear predictor that combines several linear predictors. This provides a natural extension of Cox model that leads to a mixture hazards model. We investigate the property of the maximum likelihood estimator for the proposed model. Moreover, we propose two strategies for getting the interpretable estimates. The first is to restrict the model structure in advance, based on unsupervised learning or prior information, and the second is to obtain as parsimonious an expression as possible in the parameter estimation strategy with cross- L_1_ penalty. The performance of the proposed method are evaluated by simulation and application studies.

**Results:**

We showed that the maximum likelihood estimator has consistency and asymptotic normality, and the cross- L_1_-regularized estimator has root-*n* consistency. Simulation studies show these properties empirically, and application studies show that the proposed model improves predictive ability relative to Cox model.

**Conclusions:**

It is essential to capture the intrinsic heterogeneity of patients for getting more stable and effective risk score. The proposed hazard model can capture such heterogeneity and achieve better performance than the ordinary linear Cox proportional hazards model.

## Background

Medical science has made dramatic progress in recent years and reached the stage of trying to develop treatment tailored to patients’ individual characteristics. In particular, it is becoming standard for doctors to prescribe selective therapeutic agents to cancer patients with specific oncogenes. Such personalized medicines are not only effective, but also economical and practical: if personalization fulfills its promise, patients no longer have to try expensive but ineffective treatments, or suffer from unnecessary side effects.

The idea of treatment individualization arose from the fact that patients often show different responses, in terms of both therapeutic and side effects, to the same specific treatments. Therefore, in order to realize individualized treatment, it is necessary to predict treatment risk accurately and carefully based on patients’ characteristics. Because such a prediction should be performed in an objective manner, we need some quantified measurement of risk. This is usually achieved by a risk score, estimated by a regression model derived from various types of datasets. Survival time datasets are among the most popular source of data in medical science because they focus on extension of time-to-event (in this case, an undesirable or bad event). Several types of events can be considered, including cancer prognosis or metastasis, myocardial infarction, and death. For time-to-event data with right-censoring, it is standard to apply the relative risk model. The main feature of the model is the assumption that hazards are proportional; i.e., the hazard ratio of two different subjects depends only on their covariates. Despite requiring this somewhat strong assumption, this type of model is used in a broad range of applications. Although the relative risk model covers a wider range, the exponential relative risk model, known as Cox’s proportional hazards model, is most common for these applications. For simplicity, in the rest of this paper we assume that all covariates are time-independent and that there are no event ties, but these assumptions can be relaxed. In the Cox model, it is assumed that the log hazard is decomposed into and time-independent linear predictor of covariates vector ***x*** as log(*h*(*t*|***x***)/*h*_0_(*t*))=***β***^⊤^***x***, where *h*(*t*|***x***) is a hazard rate at time *t*, *h*_0_(*t*)=*h*(*t*|***0***) is called the baseline hazard function, and ***β*** is a coefficient vector.

However, the relationship between the hazard and covariates may not be common among subjects. For example, recent clinical studies focused on the fact that heterogeneity among such populations can result in different responses to the same treatment. Such complex relationships can no longer be described in a single hazard model. Therefore, we should consider a mixture hazard model to capture the different hazard patterns in the heterogeneous population. In fact, [1] and [2] introduced a general family of mixture hazard models to describe multimodal hazards, although these models were described in a limited situation under a parametric approach. Also, in the context of a cure model, a binary hazard mixture model was proposed in a semi-parametric manner [3]. In this paper, we propose a natural extension of Cox’s proportional hazards model using a quasi-linear predictor that leads to a proportional model with mixture hazards.

The rest of the article is organized as follows. In “Methods” section, we derive the mixture hazard model via the quasi-linear predictor and two strategies are developed to obtain parsimonious expression: the restricted quasi-linear model and the cross- L_1_-penalty estimation. In “Results” section, we investigate the estimators’ asymptotic properties. Moreover, we present numerical simulations and applications to real data sets, respectively. The proofs for all propositions and theorems given as Appendix are available in Additional file 1.

## Methods

### Quasi-linear Cox Model

#### Formulations

Let *t* be the survival time of a subject with baseline covariate vector ***x***. Then we define the quasi-linear Cox model as

1 $$  h(t|\boldsymbol{x},\boldsymbol{\pi}, \boldsymbol{\beta})= h_{0}(t)\exp{(f_{Q}(\boldsymbol{x}, \boldsymbol{\pi}, \boldsymbol{\beta}))}.  $$

Here, *f*_*Q*_ is a quasi-linear predictor function defined by the log-sum- exp averages of *K* linear predictors [4] as

2 $$ f_{Q}\left(\boldsymbol{x}, \boldsymbol{\pi}, \boldsymbol{\beta}\right) = \log \left(\sum_{k=1}^{K} \pi_{k} \exp{\left(\boldsymbol{\beta}_{k}^{\top}\boldsymbol{x}\right)}\right),  $$

where ***π***^⊤^=(*π*_1_,⋯,*π*_*K*_) is a vector of mixing proportion with $\sum _{k=1}^{K} \pi _{k} = 1$, and $\boldsymbol {\beta }^{\top }=(\boldsymbol {\beta }_{1}^{\top }, \cdots, \boldsymbol {\beta }_{K}^{\top })$ is the coefficient vector. The relationship between hazard and covariates differs among subpopulations; the parameter *K* relies on the total number of subpopulations that satisfy this condition. We find that the quasi-linear Cox model can be understood as a mixture hazard model because from (1) and (2)

3 $$ h\left(t|\boldsymbol{x},\boldsymbol{\pi}, \boldsymbol{\beta}\right)=\sum_{k=1}^{K} \pi_{k} h_{0}(t)\exp\left(\boldsymbol{\beta}_{k}^{\top} \boldsymbol{x}\right).  $$

The underlying hazard model in (3) is described as $h(t|\boldsymbol {x},\boldsymbol {\pi }, \boldsymbol {\beta }) = \sum _{k=1}^{K} \pi _{k} h_{k}(t|\boldsymbol {x}, \boldsymbol {\beta }_{k}),$ where $h_{k} (t|\boldsymbol {x},\boldsymbol {\beta }_{k}) = h_{0k}(t) \exp (\boldsymbol {\beta }_{k}^{\top } \boldsymbol {x})$ with the assumption that *h*_0*k*_(*t*)=*h*_0_(*t*) for any *k*. Thus the proposed model can be understood as the special case of the mixture of Cox’s proportional hazards models. The assumption about equality of the baseline hazard function may seem somewhat stronger, but this simplifies the model formulation and interpretability. Simulations and application studies in “Simulations” section and “Application” section show that model (3) has sufficient predictive ability. A more general model that removes the assumption of equality of the baseline hazard function is discussed in the “Discussion” section.

### Partial likelihood and maximum likelihood estimator

Consider the data (***x***_*i*_,*t*_*i*_,*δ*_*i*_) (*i*=1,2,⋯,*n*) from *n* subjects, where ***x***_*i*_ is a *p*-dimensional covariates vector, *t*_*i*_ is observed survival or censored time, and *δ*_*i*_ is an event indicator which takes a value of 1 if the sample experiences the event by *t*=*t*_*i*_ and 0 otherwise. We assume that *t*_*i*_ and *δ*_*i*_ are independent for all subjects. Let ***θ***^⊤^=(***π***^⊤^,***β***^⊤^) and $\boldsymbol {\theta }_{k}^{\top }=({\pi }_{k},\boldsymbol {\beta }_{k}^{\top })$ for any *k*. Then, the partial log-likelihood function of the parameter ***θ*** is written as

4 $$ \begin{aligned} l(\boldsymbol{\theta})=\sum_{i=1}^{n} \delta_{i} \left\{\log \left(\sum_{k=1}^{K} \eta_{i}(\boldsymbol{\theta}_{k}) \right)-\log \left(\sum_{\ell \in R(t_{i})} \sum_{k=1}^{K} \eta_{\ell}(\boldsymbol{\theta}_{k})\right)\right\}, \end{aligned}  $$

where *R*(*t*_*i*_)={*l*∈{1,⋯,*n*}|*t*_*i*_≤*t*_*ℓ*_} and $\eta _{i}(\boldsymbol {\theta }_{k})=\pi _{k} \exp {(\boldsymbol {\beta }_{k}^{\top } \boldsymbol {x}_{i})}$. The *R*(*t*_*i*_) denotes the risk set at time *t*_*i*_. The maximum partial likelihood estimator $\hat {\boldsymbol {\theta }}$ of (4) has consistency and asymptotic normality, as shown in “Asymptotic properties” section. Because we cannot get the estimates analytically, as with the Cox’s proportional hazards model, we need some numerical optimization method. As an example of such a method, the outline of the Minorization-Maximization (MM) algorithm [5] is shown here. The convergence property of the algorithm is demonstrated in Appendix A.

First, the score function of the partial likelihood (4) consists of the following elements:

5 $$\begin{array}{*{20}l} \frac{\partial}{\partial\pi_{m}}l(\boldsymbol{\theta}) &= \sum_{i=1}^{n} \delta_{i} \left(\frac{p_{mi}(\boldsymbol{\theta}) - p^{*}_{mi}(\boldsymbol{\theta})}{\pi_{m}}\right) \end{array} $$

and

6 $$ {}{\begin{aligned} \frac{\partial}{\partial \boldsymbol{\beta}_{m}}l(\boldsymbol{\theta}) &= \sum_{i=1}^{n} \delta_{i} \left(p_{mi}(\boldsymbol{\theta}) x_{i} - p^{*}_{mi}(\boldsymbol{\theta}) \frac{ \sum_{\ell \in R(t_{i})} \eta_{\ell}(\boldsymbol{\theta}_{m}) x_{l} }{\sum_{\ell \in R(t_{i})} \eta_{\ell}(\boldsymbol{\theta}_{m}) }\right)\!, \end{aligned}}  $$

where

$$ p_{mi}(\boldsymbol{\theta}) = \frac{\eta_{i}(\boldsymbol{\theta}_{m})}{\sum_{k=1}^{K} \eta_{i}(\boldsymbol{\theta}_{k})}= \frac{\pi_{m}\exp\left(\boldsymbol{\beta}_{m}^{\top} x_{i}\right)}{\sum_{k=1}^{K} \pi_{k} \exp\left(\boldsymbol{\beta}_{k}^{\top} x_{i}\right)}, $$

$$ \begin{aligned} p_{mi}^{*}(\boldsymbol{\theta}) &= \frac{\sum_{\ell \in R(t_{i})} \eta_{\ell}(\boldsymbol{\theta}_{m})}{\sum_{\ell \in R(t_{i})} \sum_{k=1}^{K} \eta_{\ell}(\boldsymbol{\theta}_{k})} \\&= \frac{\sum_{\ell \in R(t_{i})} \pi_{m}\exp\left(\boldsymbol{\beta}_{m}^{\top} x_{i}\right)}{\sum_{\ell \in R(t_{i})} \sum_{k=1}^{K} \pi_{k} \exp\left(\boldsymbol{\beta}_{k}^{\top} x_{i}\right)}. \end{aligned} $$

We remark that the function *l*(***θ***) is rather complicated relative to the one of Cox’s model, which typically contains summands in the logarithmic function. In fact, when *K*=1, (4) is reduced to the standard form of the partial log-likelihood function *l*(***β***_1_) in which the gradient vector becomes

7 $$\begin{array}{*{20}l}  \frac{\partial}{\partial \boldsymbol{\beta}_{1}}l(\boldsymbol{\beta}_{1}) & = \sum_{i=1}^{n} \delta_{i}\left(\boldsymbol{x}_{i} - \frac{\sum_{\ell \in R(t_{i})} \exp\left(\boldsymbol{\beta}_{1}^{\top} \boldsymbol{x}_{\ell}\right)\boldsymbol{x}_{\ell} }{\sum_{\ell \in R(t_{i})} \exp\left(\boldsymbol{\beta}_{1}^{\top} \boldsymbol{x}_{\ell}\right)}\right). \end{array} $$

Compared with (7), repeated calculation of (5) and (6) is computationally hard. We therefore consider a simpler function as

$$ \begin{aligned} {}G(\boldsymbol{\theta},\boldsymbol{\theta}_{0}) &= l(\boldsymbol{\theta}_{0}) + \sum_{i=1}^{n}\sum_{k=1}^{K} \delta_{i} p_{ki}(\boldsymbol{\theta}_{0})\log \frac{\pi_{k}\exp\left(\boldsymbol{\beta}_{k}^{\top} \boldsymbol{x}_{i}\right)}{\pi_{0k}\exp\left(\boldsymbol{\beta}_{0k}^{\top} \boldsymbol{x}_{i}\right)} \\  &- \sum_{i=1}^{n} \delta_{i} \left\{\frac{\sum_{\ell\in R(t_{i})}\sum_{k=1}^{K} \pi_{k} \exp\left(\boldsymbol{\beta}_{k}^{\top} \boldsymbol{x}_{\ell}\right)} {\sum_{\ell\in R(t_{i})} \sum_{k=1}^{K}\pi_{0k}\exp\left(\boldsymbol{\beta}_{0k}^{\top} \boldsymbol{x}_{\ell}\right)}-1\right\}, \end{aligned}  $$

which has only feasible terms of log hazard and log cumulative hazard functions. Thus, we observe that

8 $$ \begin{aligned} \frac{\partial}{\partial\pi_{m}} G(\boldsymbol{\theta},\boldsymbol{\theta}_{0}) &= \sum_{i=1}^{n} \delta_{i} \left\{\frac{ p_{mi}(\boldsymbol{\theta}_{0})}{\pi_{m}}-\frac{p_{mi}^{*}(\boldsymbol{\theta}_{0})}{\pi_{m}}\frac{\sum_{\ell\in R(t_{i})} \pi_{m}\exp\left(\boldsymbol{\beta}_{m}^{\top} \boldsymbol{x}_{\ell}\right)}{\sum_{\ell\in R(t_{i})} \pi_{0m}\exp\left(\boldsymbol{\beta}_{0m}^{\top} \boldsymbol{x}_{\ell}\right)} \right\} \end{aligned}  $$

and

9 $$\begin{array}{*{20}l} \frac{\partial}{\partial\boldsymbol{\beta}_{m}} G(\boldsymbol{\theta},\boldsymbol{\theta}_{0}) &= \sum_{i=1}^{n} \delta_{i} \left\{{\vphantom{\frac{\sum_{\ell\in R(t_{i})} \pi_{m} \exp\left(\boldsymbol{\beta}_{m}^{\top} \boldsymbol{x}_{\ell}\right)\boldsymbol{x}_{\ell}}{{\sum_{\ell \in R(t_{i})}\pi_{0m}\exp\left(\boldsymbol{\beta}_{0m}^{\top} \boldsymbol{x}_{\ell}\right)}}}} p_{mi}(\boldsymbol{\theta}_{0}) \boldsymbol{x}_{i}\right. \\&-\left. p_{mi}^{*}(\boldsymbol{\theta}_{0}) \frac{\sum_{\ell\in R(t_{i})} \pi_{m} \exp\left(\boldsymbol{\beta}_{m}^{\top} \boldsymbol{x}_{\ell}\right)\boldsymbol{x}_{\ell}}{{\sum_{\ell \in R(t_{i})}\pi_{0m}\exp\left(\boldsymbol{\beta}_{0m}^{\top} \boldsymbol{x}_{\ell}\right)}} \right\}, \end{array} $$

which leads to $\frac {\partial }{\partial \boldsymbol {\theta }}l(\boldsymbol {\theta })= \frac {\partial }{\partial \boldsymbol {\theta }} G(\boldsymbol {\theta },\boldsymbol {\theta }_{0})|_{\boldsymbol {\theta }_{0}=\boldsymbol {\theta }}.$ Exploring these properties, we propose a learning algorithm $\left \{\boldsymbol {\theta }^{(s)}=(\boldsymbol {\pi }^{(s)},\boldsymbol {\beta }^{(s)})| s \in {\mathcal {S}}\right \}$ for the maximum partial likelihood estimator of ***θ*** by sequential maximization of *G*(***θ***,***θ***_0_) as ***θ***^(*s*+1)^=argmax_***θ***_*G*(***θ***,***θ***^(*s*)^) for all *s* in ${\mathcal {S}}= \{1, 2, \cdots, S\}$, where ***θ***^(1)^ is an initial value and *S* denotes a stopping time. By definition, we obtain *G*(***θ***^(*s*+1)^,***θ***^(*s*)^)≥*G*(***θ***^(*s*)^,***θ***^(*s*)^). It follows from (8) and (9) that the iteration step is given by $\boldsymbol {\theta }^{(s+1)}=\left (\tilde {\pi }_{k}\left (\boldsymbol {\theta }^{(s)}\right),\tilde {\boldsymbol {\beta }}_{k}\left (\boldsymbol {\theta }^{(s)}\right)\right)$, where

10 $$\begin{array}{*{20}l} {}\tilde{\pi}_{k}(\boldsymbol{\theta}) &= \frac{1}{z(\boldsymbol{\theta},\tilde{\boldsymbol{\beta}}_{k}(\boldsymbol{\theta}^{(s)}))} \frac{\sum_{i=1}^{n} \delta_{i} p_{ki}(\boldsymbol{\theta})} {\sum_{i=1}^{n} \delta_{i} p_{ki}^{*}(\boldsymbol{\theta}) \frac{\sum_{\ell \in R(t_{i})} \exp\left(\tilde{\boldsymbol{\beta}}_{k}^{\top} \boldsymbol{x}_{\ell}\right)}{\sum_{\ell \in R(t_{i})} \exp\left(\boldsymbol{\beta}_{k}^{\top} \boldsymbol{x}_{\ell}\right)} }\pi_{k}, \end{array} $$

11 $$ \begin{aligned} \tilde{\boldsymbol{\beta}}_{k}(\boldsymbol{\theta})&= \text{argsolve}_{\boldsymbol{b}_{k}|\boldsymbol{\theta}} \left\{ \sum_{i=1}^{n} \delta_{i} p_{ki}(\boldsymbol{\theta})\boldsymbol{x}_{i}\right.\\&=\left.\sum_{i=1}^{n}\delta_{i} p_{ki}^{*}(\boldsymbol{\theta}) \frac{\sum_{\ell \in R(t_{i})} \exp\left(\boldsymbol{b}_{k}^{\top} \boldsymbol{x}_{\ell}\right)\boldsymbol{x}_{\ell} }{\sum_{\ell \in R(t_{i})} \exp\left(\boldsymbol{\beta}_{k}^{\top} \boldsymbol{x}_{\ell}\right)} \right\}, \end{aligned}  $$

where

$${}z\left(\boldsymbol{\theta},\tilde{\boldsymbol{\beta}}_{k}\left(\boldsymbol{\theta}^{(s)}\right)\right)=\sum_{k=1}^{K} \frac{\sum_{i=1}^{n} \delta_{i} p_{ki}(\boldsymbol{\theta})} {\sum_{i=1}^{n} \delta_{i} p_{ki}^{*}(\boldsymbol{\theta}) \frac{\sum_{\ell \in R(t_{i})} \exp\left(\tilde{\boldsymbol{\beta}}_{k}^{\top} \boldsymbol{x}_{\ell}\right)}{\sum_{\ell \in R(t_{i})} \exp\left(\boldsymbol{\beta}_{k}^{\top} \boldsymbol{x}_{\ell}\right)} }\pi_{k}. $$

We observe that estimating equation in (11) is a weighted variant of standard partial likelihood equation. Furthermore, we observe that the minus Hessian matrix of *G*(***θ***,***θ***_0_) with respect to ***θ*** is positive-definite, which guarantees that $\boldsymbol {\theta }^{(s+1)}=\left (\tilde {\pi }_{k}\left (\boldsymbol {\theta }^{(s)}\right),\tilde {\boldsymbol {\beta }}_{k}\left (\boldsymbol {\theta }^{(s)}\right)\right)$, as discussed above, is the unique minimizer of *G*(***θ***,***θ***^(*s*)^) in ***θ***. We observe the basic property of the learning algorithm, $\left \{\boldsymbol {\theta }^{(s)}=\left (\pi ^{(s)},\boldsymbol {\beta }^{(s)}\right)\big | s \in {\mathcal {S}}\right \}$, as follows.

#### **Proposition 1**

Let $\left \{\boldsymbol {\theta }^{(s)}=\left (\pi ^{(s)},\boldsymbol {\beta }^{(s)}\right)\big | s \in {\mathcal {S}}\right \}$ be the fixed-point algorithm defined by the iteration rules (*10*) and (*11*). Then, the partial log-likelihood function *l*(***θ***) increases on the sequence $\left \{\boldsymbol {\theta }^{(s)} | s \in {\mathcal {S}}\right \}$ as *l*(***θ***^(*s*+1)^)≥*l*(***θ***^(*s*)^) for any *s*=1,⋯,*S*−1.

The proof of Proposition 1 is given in Appendix B. The convergence of the algorithm {***θ***^(*s*)^:*s*≥1} to the maximum partial likelihood estimator $\hat {\boldsymbol {\theta }}$ is not directly connected to Proposition 1. We need to make some assumption about the model in order to guarantee convergence, similar to that of expectation–maximization (EM) algorithm [6] for the analytic conditions. For example, we assume that *l*(***θ***) is unimodal, with ***θ***^∗^ being the only stationary point. We note that *∂G*(***θ***,***θ***_0_)/*∂****θ*** is continuous for ***θ*** and ***θ***_0_. Thus, the sequence {***θ***^(*s*)^} converges to the unique maximizer ***θ***^∗^ [7]. In fact, the partial likelihood function *l*(***θ***) is expressed as a difference of two concave functions *ψ*_1_(***θ***) and *ψ*_2_(***θ***), where $\psi _{1}(\boldsymbol {\theta }) = -\sum _{i=1}^{n} \delta _{i} \log \sum _{k=1}^{K} \pi _{k} \exp \left (\boldsymbol {\beta }_{k}^{\top } \boldsymbol {x}_{i}\right)$ and $\psi _{2}(\boldsymbol {\theta }) = -\sum _{i=1}^{n} \delta _{i} \log \sum _{j\in R(t_{i})} \sum _{k=1}^{K} \pi _{k} \exp \left (\boldsymbol {\beta }_{k}^{\top } \boldsymbol {x}_{j}\right)$. Hence, the assumption for the unimodality is necessary for convergence.

### Parsimonious Model

**Fig. 1:**
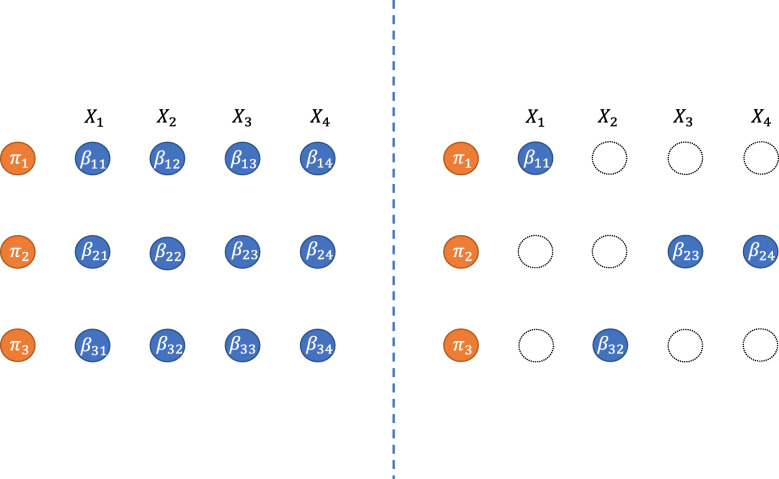
The conceptual diagram of the full and parsimonious models in the setting of *p*=4 and *K*=3 are drawn. The left figure shows the full model by the quasi-linear predictor *f*_*Q*_. The right figure shows the parsimonious model written in the same predictor but has some zero-coefficients

The quasi-linear Cox model consists of a relatively large number of parameters. Moreover, each covariate has multiple roles in every linear predictor. These complexities compromise the stability of parameter estimation and the interpretability of the model overall. Accordingly, we need a more parsimonious expression as shown in Fig. 1. To obtain a more parsimonious model, we propose a variant of the proposed model and parameter estimation procedure: restricted quasi-linear Cox model and cross- L_1_-penalty method. The former idea relies on restricting the model structure in advance based on prior information. If there is prior knowledge that some factors strongly depends on the hazard of subpopulation and weakly on the hazards of other subpopulations, then we use the restricted quasi-linear form to insert the knowledge into consideration. If this is not the case, then a penalty is needed to bring full model (3) closer to the parsimonious model (13). We achieve this by using cross- L_1_ penalty introduced in “Quasi-linear Cox model with cross L_1_ penalty” section.

#### Restricted quasi-linear Cox model

The first strategy is to use the idea of disjoint sets of covariates, as proposed by [4]. In the strategy, we assume that we know the disjoint decomposition of ***x***_*i*_ as ***x***_*i*(1)_,⋯,***x***_*i*(*K*)_ with a fixed group size *K*, and that this is identical among individuals. We denote the size of ***x***_*i*(*k*)_ as *p*_*k*_, where $\sum _{k=1}^{K} p_{k}=p$. We note that such decomposition is given by prior knowledge about the disjoint structure of ***x***. The disjoint sets of covariates yield the restricted quasi-linear predictor defined by

12 $$ \begin{aligned} f_{Q}^{Res}\left(\boldsymbol{x}_{(1)},\boldsymbol{x}_{(2)},\cdots,\boldsymbol{x}_{(K)},\boldsymbol{\pi},\boldsymbol{\beta}\right) \,=\, \log \left(\sum_{k=1}^{K} \pi_{k} \exp(\boldsymbol{\beta}_{k}^{\top} \boldsymbol{x}_{(k)}) \right). \end{aligned}  $$

The mixture hazard model (1) is modified by replacing *f*_*Q*_ with $f_{Q}^{Res}$ as

13 $$\begin{array}{*{20}l}  &h^{Res}\left(t|\boldsymbol{x}_{(1)},\cdots,\boldsymbol{x}_{(K)},\boldsymbol{\pi}, \boldsymbol{\beta}\right)\\&\quad=h_{0}(t)\exp{\left(f_{Q}^{Res}\left(\boldsymbol{x}_{(1)},\cdots,\boldsymbol{x}_{(K)}, \boldsymbol{\pi}, \boldsymbol{\beta}\right)\right)}. \end{array} $$

The maximum partial likelihood estimator is calculated by the fixed-point algorithm proposed for the non-restricted version, with easy modifications.

#### Quasi-linear Cox model with cross L_1_ penalty

In the second strategy, we regularize the log-likelihood function by cross- L_1_ penalty defined by

14 $$\begin{array}{*{20}l} P_{c}(\boldsymbol{\beta})=n \lambda_{c} \sum_{\ell \neq m} \sum_{j=1}^{p} \frac{|\beta_{\ell j} \beta_{m j}|}{|\hat{\beta}_{\ell j} \hat{\beta}_{m j}|},  \end{array} $$

where *λ*_*c*_ is a regularization parameter and *β*_*kj*_ and $\hat {\beta }_{kj}$ are the *j*-th component of *k*-th coefficient vector ***β***_*k*_ and corresponding maximum partial likelihood estimator, respectively. When *λ*_*c*_ goes to infinity, the estimated parameter of the ***β***_*k*_’s would be cross-sparse; if *β*_*ℓj*_≠0, then *β*_*kj*_=0 for any *k*≠*ℓ*, and the estimated model belongs to the class of restricted quasi-linear models (13). We define the regularized log-likelihood function with cross- L_1_ penalty as

15 $$\begin{array}{*{20}l} l^{pen}(\boldsymbol{\theta})= l(\boldsymbol{\theta})-P_{2}(\boldsymbol{\beta})-P_{c}(\boldsymbol{\beta}),  \end{array} $$

where *P*_2_(***β***)=*nλ****β***^⊤^***β***, known as the L_2_ penalty. We note that an additional regularization factor such as L_1_ penalty yields the elastic net-type regularization *l*^*pen*∗^(***θ***)=*l*(***θ***)−*νP*_1_(***β***)−(1−*ν*)*P*_2_(***β***)−*P*_*c*_(***β***). We refer to the maximizer $(\boldsymbol {\tilde {\pi }},\boldsymbol {\tilde {\beta }})$ of *l*^*pen*^(***θ***) as *CLASSO* (Cross least absolute shrinkage and selection operator) estimator. The penalty (14) is a variant of adaptive L_1_ penalty originally introduced by [8]. The adaptive weights $|\hat {\beta }_{\ell j} \hat {\beta }_{m j}|$ are needed to equip *CLASSO* estimator with root-*n* consistency (see Theorem 2 in “Asymptotic properties” section). We make the following proposition regarding the *CLASSO* estimator.

##### **Proposition 2**

Let ${\mathcal {R}}_{c}$ be a region in $\mathbb {R}^{pK}$ defined as ${\mathcal {R}}_{c} = \left \{\boldsymbol {\beta } \in \mathbb {R}^{pK} | P_{c}(\boldsymbol {\beta })\leq c, P_{2}(\boldsymbol {\beta }) \leq c\right \}$. Then the region ${\mathcal {R}}_{c}$ is a convex set.

Due to the convexity of the sum of cross- L_1_ and - L_2_ penalties, the *CLASSO* estimator also has consistency and asymptotic normality as shown in Theorem 2. Empirically, however, we do not need to regularize the partial log-likelihood by *P*_2_(***β***) for stable estimation. Therefore, we consider only the *CLASSO* penalty in the empirical studies in the Simulations and Applications sections.

To get the *CLASSO* estimator, we use the full gradient algorithm [9] in the updating step for each ***β***_*k*_ (9). For each *s*-th iteration, we need to update ***β***^(*s*−1)^ to get ***β***^(*s*)^ by gradient algorithm. Let *S*_*s*_ be the stopping time in the *s*-th iteration step. The initial value $\boldsymbol {\beta }_{k}^{(s+1,0)}=\boldsymbol {\beta }_{k}^{(s,S_{s})}$ is repeatedly updated as

16 $$ \begin{aligned} \boldsymbol{\beta}_{m}^{(s+1,u+1)} &= \boldsymbol{\beta}_{m}^{(s+1,u)}+\text{min} \left\{t_{\text{opt}}(\boldsymbol{\pi}^{(s)},\boldsymbol{\beta}^{(s+1,u)}), t_{\text{edge}}(\boldsymbol{\pi}^{(s)},\right.\\&\quad\left.\boldsymbol{\beta}^{(s+1,u)})\right\}\boldsymbol{d}_{m}\left(\boldsymbol{\pi}^{(s)},\boldsymbol{\beta}^{(s+1,u)}\right), \end{aligned}  $$

where

$$\begin{array}{*{20}l} {}\boldsymbol{d}_{m}(\boldsymbol{\theta})&=(d_{m1} (\boldsymbol{\theta}),d_{m2} (\boldsymbol{\theta}),\cdots,d_{mp} (\boldsymbol{\theta}))^{\top},  \end{array} $$

$$\begin{aligned} t_{\text{edge}}(\boldsymbol{\theta})\,=\,\min_{1 \leq j \leq p}\left(-\frac{\beta_{mj}}{d_{mj} (\boldsymbol{\theta})} : \text{sign}(\beta_{mj})=-\text{sign}(d_{mj}(\boldsymbol{\theta}))\neq 0\right),\end{aligned} $$

and

$$t_{\text{opt}}(\boldsymbol{\theta})=\frac{|d_{m}(\boldsymbol{\theta})|}{d_{m}(\boldsymbol{\theta})^{\top} \left\{ \frac{\partial^{2} l(\boldsymbol{\theta})}{\partial \boldsymbol{\beta} \partial \boldsymbol{\beta}^{\top}} \right\}d_{m}(\boldsymbol{\theta})}.$$

Here

17 $$ \begin{aligned} d_{mj}(\boldsymbol{\theta})= \left\{\begin{array}{ll} &{}\frac{\partial G(\boldsymbol{\theta},\boldsymbol{\theta}^{(s)})}{\partial \beta_{mj}} -\lambda_{c} \left({\sum_{k \neq m}}{|\beta_{kj}|}\right) \text{sign}(\beta_{mj}) \ \ \ \ \ \ \text{if}\ \beta_{mj} \neq 0\\ &{}\frac{\partial G(\boldsymbol{\theta},\boldsymbol{\theta}^{(s)})}{\partial \beta_{mj}} -\lambda_{c} \left({\sum_{k \neq m}}{|\beta_{kj}|}\right)\text{sign}\left(\frac{\partial G(\boldsymbol{\theta},\boldsymbol{\theta}^{(s)})}{\partial \beta_{mj}}\right) \ \ \ \text{if}\ \beta_{mj}=0, \ \ \ \\&{}\left| \frac{\partial G\left(\boldsymbol{\theta},\boldsymbol{\theta}^{(s)}\right)}{\partial \beta_{mj}}\right|>\lambda_{c} \left({\sum_{k \neq m}}{|\beta_{kj}|}\right) \ \ \ \ \\ &{}0\ \ \ \ \ \ \ \ \ \ \ \ \ \ \ \ \ \ \ \ \ \ \ \ \ \ \ \ \ \ \ \ \ \ \ \ \ \ \ \ \ \ \ \ \ \ \ \ \ \ \text{otherwise} \end{array}\right.  \end{aligned}  $$

for *j*=1,⋯,*p*, where sign(·) is a sign function defined by setting sign(*z*) equal to 1 for *z*>0, 0 for *z*=0 and −1 for *z*<0. In each step, *t*_opt_ provides the optimal solution of the gradient descent algorithm, and *t*_edge_ controls the direction of the gradient so as not to change the signs of parameters.

For all analysis in Simulations and Applications sections, the initial values of parameters were set to the equal probability weighting parameters *π*_*k*_=1/*K* for *k*=1,2,⋯,*K* and the coefficient vectors of Cox’s proportional hazard models estimated from random *K*-samples sets on the parameter estimation of the quasi-linear Cox model. The tuning parameter *λ*_*c*_ is determined by any model selection criteria such as AIC or estimated test AUC from bootstrap estimates other than BIC. In this paper, we use Bayes Information Criteria (BIC) [10] because (i) it is one of the most popular information criteria, (ii) it is computationally easy to calculate compared with the estimator which relies on the bootstrap sampling and (iii) it has the consistency in model selection.

## Results

### Asymptotic properties

In this section, we provide an asymptotic property of the maximum likelihood estimator $\boldsymbol {\hat {\theta }}$ and the coefficient part of *CLASSO* estimator $\boldsymbol {\tilde {\beta }}$. In the following, our discussion is based on the stochastic process. For the *i*-th individual, let $\phantom {\dot {i}\!}N_{i}(t)=1_{\{t_{i}\leq t, \delta _{i}=1 \}}(t)$ be the right-continuous counting process, where each *N*_*i*_(*t*) counts the number of observed events on (0,*t*], and let $\phantom {\dot {i}\!}Y_{i}(t)=1_{\{t_{i} \geq t, c_{i} \geq t\}}(t)$ be the left-continuous at-risk process that shows the observation status at time *t*, where *c*_*i*_ and *t*_*i*_ are censoring and true survival times. Here 1_*E*_ is an indicator function defined by setting 1_*E*_(*t*) equal to 1 for *t*∈*E* and equal to 0 for *t*∉*E*. Let us denote ${\mathcal F}_{t}=\sigma \left \{N_{i}(u),Y_{i}(u^{+});i=1,\cdots,n;0\leq u \leq t\right \}$ as the *σ*-algebra generated by all *N*_*i*_(*u*) and *Y*_*i*_(*u*),0≤*u*≤*t*. Then, the corresponding intensity process of *N*_*i*_(*t*) is defined by $\Lambda _{i}(t)dt=P(dN_{i}(t)=1|{\mathcal F}_{t_{-}})$ and the proposed model is described as

18 $$\begin{array}{*{20}l} P(dN_{i}(t)=1|{\mathcal F}_{t_{-}})=Y_{i}(t)h_{0}(t) \exp(f_{Q}(\boldsymbol{x},\boldsymbol{\theta}_{0})), \end{array} $$

where $\boldsymbol {\theta }_{0}=(\boldsymbol {\pi }_{0}^{\top }, \boldsymbol {\beta }_{0}^{\top })^{\top }$. Then we get two theorems about the maximum partial likelihood estimator and *CLASSO* estimator.

#### **Theorem 1**

Let $\hat {\boldsymbol {\theta }}=(\hat {\boldsymbol {\pi }}^{\top },\hat {\boldsymbol {\beta }}^{\top })^{\top }$ be the maximum partial likelihood estimator in the quasi-linear Cox model. Assume that the regularity conditions A-D (in Appendix C) hold. Then it follows that

1. (Consistency) $\hat {\boldsymbol {\theta }} \stackrel {p}{\longrightarrow } \boldsymbol {\theta _{0}}$
2. (Asymptotic Normality) $\sqrt {n}(\boldsymbol {\hat {\theta }}-\boldsymbol {\theta _{0}})\stackrel {d}{\longrightarrow } \mathrm {N}(\boldsymbol {0},I^{-1}(\boldsymbol {\theta }_{0}))$

#### **Theorem 2**

Let $\tilde {\boldsymbol {\theta }}=(\tilde {\boldsymbol {\pi }}^{\top },\tilde {\boldsymbol {\beta }}^{\top })^{\top }$ be the *CLASSO* estimator in the quasi-linear Cox model with cross L_1_ penalty. Assume that condition A-D (in Appendix C) hold. If $\sqrt {n}\lambda _{n} \rightarrow \infty $, then $||\tilde {\boldsymbol {\beta }}- \boldsymbol {\beta }_{0}|| = O_{p} (n^{-1/2})$.

Theorem 1 shows that the partial maximum likelihood estimator has the consistency and asymptotic normality. Theorem 2 shows that by choosing a proper sequence *λ*_*n*_, there exists a $\sqrt {n}$-consistent *CLASSO* estimator. The proofs are given in Appendix C and D.

### Simulations

#### Settings

We conducted the simulations described in this section with two objectives in mind. The first was to ascertain whether the consistency of the maximum partial-likelihood estimator can be observed empirically. The second was to ascertain whether tuning parameter *λ*^(*c*)^ selection can be performed efficiently using the BIC.

In all simulation studies introduced here, the inverse function method was used for data generation. First, the covariates ***x*** were generated from the multivariate normal distribution, the apparent censored time *T*_1_ was generated from the exponential distribution with mean 1000, and the random variable *U* was generated from the uniform distribution on [0,1]. Let the baseline survival time be followed the exponential distribution with mean 100. Then, the true survival time corresponding to the log relative risk function $f_{Q}^{Res}(\boldsymbol {x}; \boldsymbol {\theta })$ was given as $T_{2} = -(\log (U)/100)\exp \left (f_{Q}^{Res}(\boldsymbol {x}; \boldsymbol {\theta })\right)$. Based on *T*_1_ and *T*_2_, let *T*= min(*T*_1_,*T*_2_) be the observational survival time and *δ*=*I*(*T*_1_<*T*_2_) be the censored indicator before the event time.

Sample size was set to *N*=400 in all scenarios, and it was assumed that the true number of the groups were known for all settings. The tuning parameter *λ* of cross- L_1_ penalty was determined by BIC. We note that the maximum candidate value of the tuning parameter *λ* was controlled sufficiently to achieve the restricted quasi-linear form for every setting. We had the following options:

- *Independent Setting (IS) or Dependent Setting (DS)*
  Each covariate vector ***x***_*i*_ was sampled from the standard normal distribution N(***0***_*p*_,*Σ*), where $\boldsymbol {a}_{p} = (a,a,\cdots,a) \in \mathbb {R}^{p}$. In IS, *Σ*=2^2^*I*_*p*_, where *I*_*p*_ is an identity matrix of size *p*. In DS, $\Sigma = (s_{ij}) \in \mathbb {R}^{p \times p}$, where *s*_*ij*_=2^2^×0.7^|*i*−*j*|^.
- *Group Size and Coefficients*
  A number of combined linear predictors, namely group size, was set to *K*=2 or *K*=3. A number of covariates, namely dimension size, was set to *p*=2,*p*=3, or *p*=5. A coefficients vector was set to cross-sparse (Scenario 1,3,5) or overlapped (Scenario 2,4,6) according to the following settings.
  1. *K*=2,*p*=2,***π***^⊤^=(0.3,0.7) and $\boldsymbol {\beta }^{\top }=(\boldsymbol {\beta }_{1}^{\top },\boldsymbol {\beta }_{2}^{\top })=((1,0),(0,1.5))$
  2. *K*=2,*p*=2,***π***^⊤^=(0.3,0.7) and $\boldsymbol {\beta }^{\top }=(\boldsymbol {\beta }_{1}^{\top },\boldsymbol {\beta }_{2}^{\top })=((1,0.5),(0,1.5))$
  3. *K*=2,*p*=5,***π***^⊤^=(0.3,0.7) and $\boldsymbol {\beta }^{\top }=(\boldsymbol {\beta }_{1}^{\top },\boldsymbol {\beta }_{2}^{\top })=((1,1,1,0,0),(0,0,0,1.5,1.5))$
  4. *K*=2,*p*=5,***π***^⊤^=(0.3,0.7) and $\boldsymbol {\beta }^{\top }=(\boldsymbol {\beta }_{1}^{\top },\boldsymbol {\beta }_{2}^{\top })=((1,1,1,0,0.5),(0,0.25,0.5,1.5,1.5))$
  5. *K*=3,*p*=3,***π***^⊤^=(0.2,0.3,0.5) and $\boldsymbol {\beta }^{\top }=(\boldsymbol {\beta }_{1}^{\top },\boldsymbol {\beta }_{2}^{\top },\boldsymbol {\beta }_{3}^{\top })=((1,0,0),(0,1.5,0),(0,0,1))$
  6. *K*=3,*p*=3,***π***^⊤^=(0.2,0.3,0.5) and $\boldsymbol {\beta }^{\top }=(\boldsymbol {\beta }_{1}^{\top },\boldsymbol {\beta }_{2}^{\top },\boldsymbol {\beta }_{3}^{\top })=((1,0.5,0),(0,1.5,0.5),(0.5,0,1))$

#### Simulation results

For each scenario, the mean values of the estimated coefficients and the mean-squared errors (MSEs) are shown in Table 1. Throughout the whole scenario, all coefficients were estimated with only a little bias. In particular, in the disjoint setting (Scenario 1,3,5 for IS and DS) we could almost certainly distinguish the zero coefficients from non-zero coefficients, indicating that BIC and cross- L_1_ penalty work well in these scenarios. Dependent situations did not have a strong effect on parameter estimation, although a slightly larger MSE was observed in comparison with the independent situations. Especially in Scenario 6 for DS, we had moderate biases in estimation of the coefficients of the first linear predictor (*β*_11_ and *β*_12_). This is because the other two groups had enough information for fitting the model to the data. In fact, the estimated risk scores between the estimated and true parameters were almost equal. This shows that the loss of model identifiability sometimes yields bias in parameter estimation for the overlapped situation; however, the predictive performance of the estimated score is sufficient.

**Table 1:** Simulation results

	Setting			*π*_1_	*π*_2_	*π*_3_		*β*_11_	*β*_12_	*β*_13_	*β*_14_	*β*_15_		*β*_21_	*β*_22_	*β*_23_	*β*_24_	*β*_25_		*β*_31_	*β*_32_	*β*_33_	
	(1)	Mean		0.31	0.69	-		1.00	0.00	-	-	-		0.00	1.52	-	-	-		-	-	-	
		MSE ×10^2^		0.34	0.34	-		0.58	0.06	-	-	-		0.07	0.84	-	-	-		-	-	-	
	(2)	Mean		0.31	0.69	-		1.01	0.49	-	-	-		0.00	1.52	-	-	-		-	-	-	
		MSE ×10^2^		1.02	1.02	-		1.10	1.04	-	-	-		0.37	0.98	-	-	-		-	-	-	
IS	(3)	Mean		0.30	0.70	-		1.01	1.00	1.00	0.00	0.00		0.00	0.00	0.00	1.50	1.50		-	-	-	
		MSE ×10^2^		0.21	0.21	-		0.02	0.01	0.01	0.67	0.53		0.41	0.51	0.42	0.00	0.00		-	-	-	
	(4)	Mean		0.31	0.69	-		0.99	0.99	0.98	-0.01	0.46		-0.01	-0.23	0.48	1.48	1.48		-	-	-	
		MSE ×10^2^		0.53	0.53	-		0.66	0.81	0.72	0.50	0.78		0.25	0.31	0.36	1.04	0.88		-	-	-	
	(5)	Mean		0.21	0.30	0.50		1.00	0.00	0.00	-	-		0.00	1.52	0.00	-	-		0.00	0.00	1.01	
		MSE ×10^2^		0.38	0.33	0.47		1.14	0.00	0.00	-	-		0.01	1.04	0.00	-	-		0.02	0.00	0.68	
	(6)	Mean		0.21	0.31	0.48		1.01	0.35	0.02	-	-		0.01	1.52	0.45	-	-		0.46	0.01	1.02	
		MSE ×10^2^		0.74	0.74	0.75		1.28	4.34	0.51	-	-		0.31	1.29	0.76	-	-		0.61	0.39	0.65	
	(1)	Mean		0.31	0.69	-		1.00	0.00	-	-	-		0.00	1.52	-	-	-		-	-	-	
		MSE ×10^2^		0.29	0.29	-		1.00	0.08	-	-	-		0.05	0.72	-	-	-		-	-	-	
	(2)	Mean		0.29	0.71	-		1.11	0.38	-	-	-		-0.01	1.53	-	-	-		-	-	-	
		MSE ×10^2^		4.33	4.33	-		9.64	9.33	-	-	-		3.64	3.90	-	-	-		-	-	-	
DS	(3)	Mean		0.30	0.70	-		1.01	1.00	1.01	0.00	0.00		0.00	0.00	0.00	1.52	1.51		-	-	-	
		MSE ×10^2^		0.22	0.22	-		0.76	1.33	1.06	0.00	0.00		0.04	0.05	0.07	0.80	0.81		-	-	-	
	(4)	Mean		0.29	0.71	-		1.00	1.00	0.99	0.00	0.43		0.01	0.22	0.47	1.50	1.48		-	-	-	
		MSE ×10^2^		0.63	0.63	-		1.12	1.83	1.82	0.77	1.82		0.34	0.71	0.82	1.09	1.10		-	-	-	
	(5)	Mean		0.20	0.29	0.51		1.03	0.00	-0.01	-	-		0.00	1.54	0.00	-	-		0.00	0.00	1.01	
		MSE ×10^2^		0.33	0.59	0.53		1.92	0.02	0.13	-	-		0.00	1.85	0.00	-	-		0.10	0.15	0.72	
	(6)	Mean		0.18	0.32	0.50		1.16	0.19	0.05	-	-		0.04	1.57	0.39	-	-		0.41	0.05	1.03	
		MSE ×10^2^		2.11	2.08	2.84		10.26	16.61	2.16	-	-		1.37	6.85	4.35	-	-		3.15	2.13	2.22	

### Application

In this section, we show the results of application studies for the breast cancer dataset in order to evaluate the performance of the quasi-linear Cox model. To evaluate the predictive ability of the learned model, we calculated the Area under the curve (AUC) of time-dependent ROC [11] using test dataset. The predictive performance was compared between Cox’s proportional hazard model and the quasi-linear Cox model. A dataset from [12] was used as the training data, and a dataset from [13] as the test data. These datasets include expression levels of 70 genes and survival time with some censors. Except for samples with missing values, there were 75 samples in the training dataset and 220 samples in the test dataset. In this application, we extracted the top 10 relevant genes to evaluate the model performance. Such marker preselection has been performed in many studies [14].

Since we had no prior knowledge for these genes, we applied the proposed model with cross- L_1_ penalty introduced in “Quasi-linear Cox model with cross L_1_ penalty” section. The number of groups *K* and the regularization parameter of the cross- L_1_ penalty *λ*_*c*_ were determined using BIC from *K*∈{2,3,4,5} and *λ*_*c*_∈{0,0.1,0.2,⋯,5.0}. All gene expressions are standardized to have mean zero and variance one among the training sample to compare the estimated coefficients. The same transformation was applied for the test sample.

**Fig. 2:**
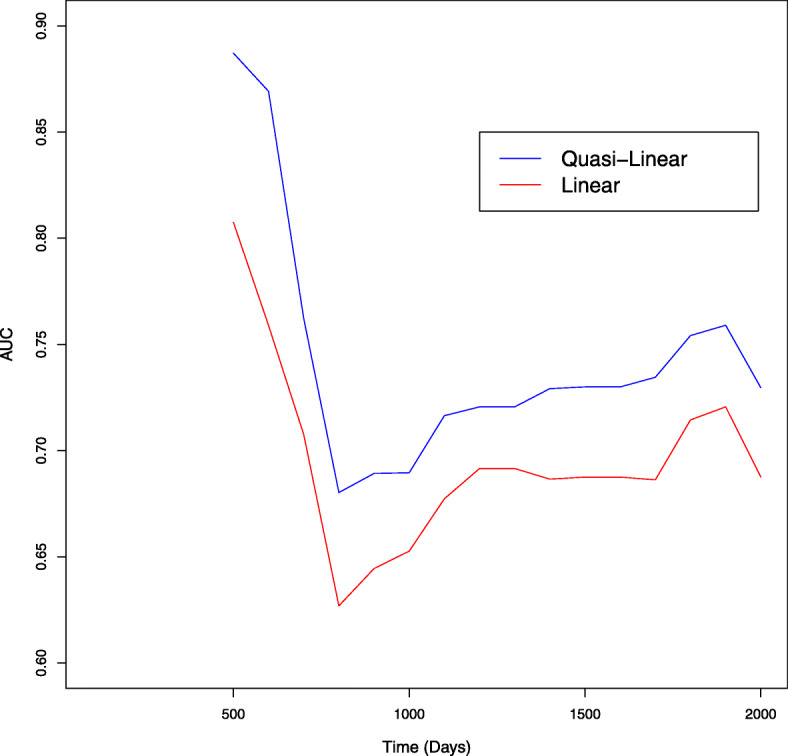
The time series changes in test AUC of the breast cancer dataset. Two line graphs show the AUC values at each time (days) calculated from time dependent ROC for the linear (red) and quasi-linear (blue) predictor

**Fig. 3:**
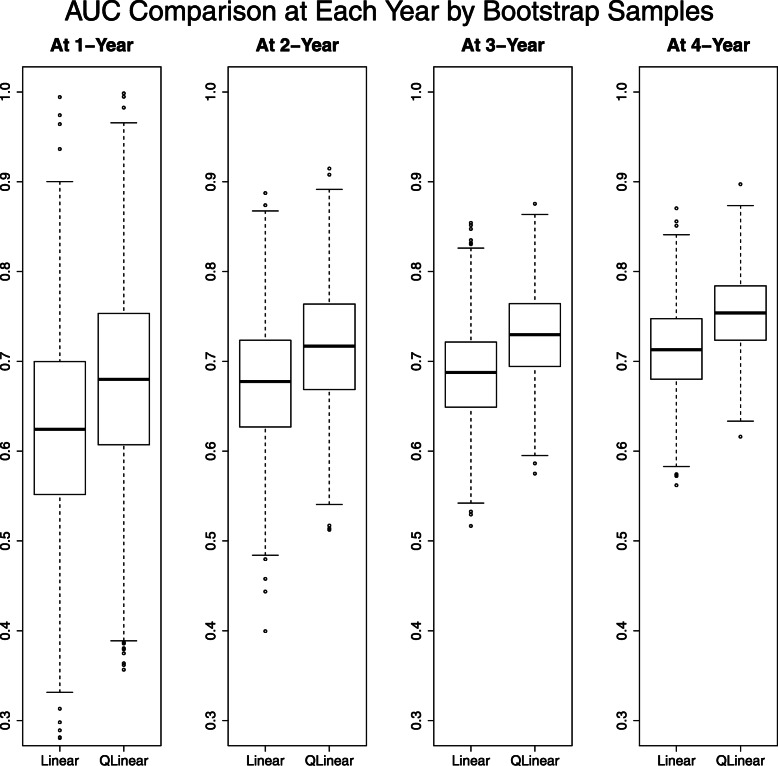
Boxplots of the test AUC by the bootstrap sample from the breast cancer dataset, where eight boxplots show the AUC values at each year (1, 2, 3 and 4) calculated from time dependent ROC for the linear (each left) and quasi-linear (each right) predictor

**Fig. 4:**
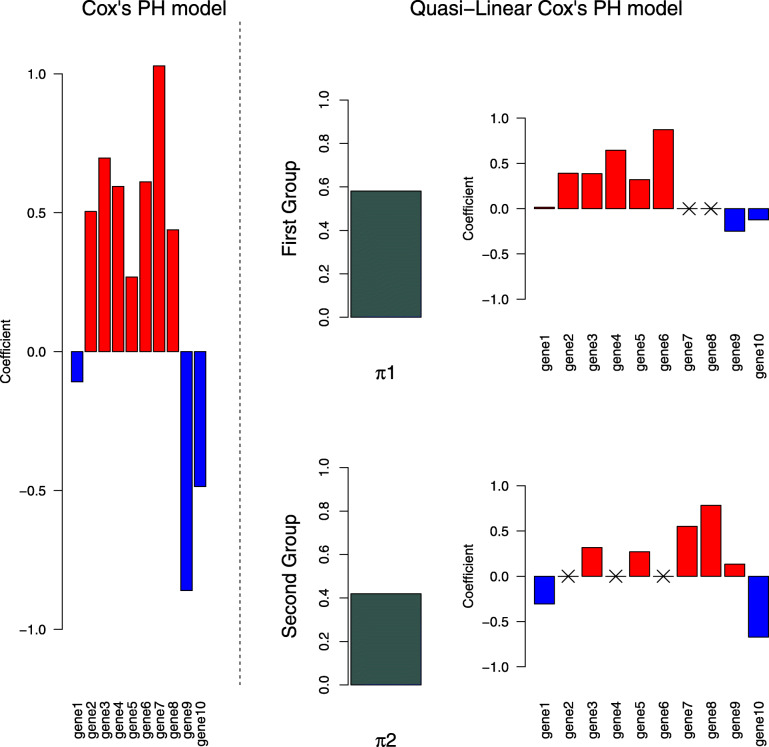
The estimated coefficients for linear and quasi-linear Cox’s proportional hazard models. The cross marks show the coefficients which equal exactly to zero

As a result, a group size *K*=2 was selected. The time series of test AUCs and the bootstrap 95% intervals for each year are shown in Figs. 2 and 3. For every time point, the test AUC of the quasi-linear relative risk model was larger than that of Cox’s proportional hazard model. The estimated coefficients for linear and quasi-linear Cox’s proportional hazard models are shown in Fig. 4. While the overall trend was not much different between linear and quasi-linear models, the cross- *L*_1_ penalty gave contrast to the fitted quasi-linear model. Five out of ten genes have zero coefficient in the hazard function in the first or second group. As a representative of them, we focus on the set of genes with the first (“gene6") and second largest (“gene4") coefficients in the first group. Those are called NUSAP1 (Nucleolar And Spindle Associated Protein 1) and TSPYL5 (Testis-Specific Y-Encoded-Like Protein 5), respectively. TSPYL5 is a well-known prognostic factor of poor outcome in breast cancer patients. Higher expression of TSPYL5 suppresses p53 protein levels causing damage to mammary cells. It may be important that those two genes have zero coefficient in the second group. Interestingly, it was reported that the TSPYL5 and NUSAP1 are biologically correlated with the same hallmarks of cancer: limitless replication potential [15]. While further investigation is needed, the proposed model thus suggests there are roughly two subpopulations which have different hazards.

## Discussion

We developed a mixture hazard model, an extension of Cox’s proportional hazards model, via a quasi-linear predictor. Theoretical discussion revealed that the maximum partial-likelihood estimator has properties of consistency and asymptotic normality. Furthermore, we showed combining the cross- L_1_ penalty makes the estimated model stable and interpretable. Empirical simulations and applications confirm these superior properties, and BIC have been shown to work well as a measure for selecting the number of groups and the tuning parameter of cross- L_1_ penalty.

We will discuss the relationship between our study and previous work. First, the quasi-linear predictor was proposed in [16] to extend the logistic regression model for capturing heterogeneous structure in biomarkers. The quasi-linear logistic model was motivated by a Bayes risk-consistent predictor in binary classification between the mixture normal distribution and single normal distributions. The quasi-linear predictor enables us to model intrinsic heterogeneity using some linear predictors, and can be used as an extension from the standard to the heterogeneous setting of several models that rely on the linear predictor. Second, as introduced in the Introduction, several studies have proposed a mixture hazard model [1, 2], but these were limited to parametric ones. Our proposed method is one extension that is possible without assuming a specific distribution. Also, several mixture distribution model proposals have been developed in past studies [17–19]. We note that the concepts of the *mixture hazards model* and *mixture density model* are completely distinct. In fact, while the mixture density model gives the simple weighted average of each survival function as the whole survival function $\tilde {S}(t)=\sum _{k=1}^{K} \tilde {\pi }_{k} \tilde {S}_{k}(t)$, the mixture hazard model gives the weighted average of log-survival function of each survival function as the whole log-survival function: $\log S(t) = \sum _{k=1}^{K} \pi _{k} \log S_{k}(t)$. In this context, the survival function is understood as the geometric mean of each survival function, $S(t)=\prod _{k=1}^{K} S_{k}^{\pi _{k}}$. We note that the density function is analogue to the probability while the hazards function is also, i.e. instantaneous rate of mortality. In this sense, both models can be regarded as the special case of latent variable models. It is not yet well understood what such a formal difference yields for the modeling in survival analysis, and it will be very important for our future work.

In this paper, we restricted the baseline hazard functions to be identical. There are three reasons for the restriction. First, it stabilizes estimation of model parameters. In addition to the high computational cost of the mixture model, it will be difficult to estimate the separate baseline hazard functions. Second, it improves the interpretability of the model. The assumption of different baseline hazards functions may seem a somewhat strange idea. This is because *baseline hazard function* refers to a hazard function when all observed covariate values are considered to be a reference for all patients. When a different baseline hazard function is required for each group, it means that there is heterogeneity that cannot be observed with the dataset in question. Instead, the quasi-linear Cox model enables us to model the intrinsic but observable heterogeneity. Third, regardless of such restrictions, the proposed model empirically had better predictive ability than the standard Cox’s proportional hazards model. We thus achieved simultaneous modeling of group-wise proportional hazards models. On the other hand, although a stratified Cox model focuses on the heterogeneity for hazards in the population with different baseline hazards, it assumes the same relative risk function among groups. These two models thus have dualistic roles to capture hazard heterogeneity in the population. Finally, we note that in theory we should be able to loose these restriction for baseline hazard function in the proposed model, based on similar ideas for density mixture models proposed by [19].

## Conclusions

In this paper, we focused on hazards mixture model. The quasi-linear Cox proportional hazards model was naturally derived by the quasi-linear predictor. It is essential to capture the intrinsic heterogeneity of patients for getting more stable and effective risk score. The proposed hazard model can capture such heterogeneity and achieve better performance than the ordinary linear Cox proportional hazards model.

## Supplementary information

**Additional file 1** Technical Derivations. In this file, we give proofs of Proposition 1, Proposition 2, Theorem 1 and Theorem 2.

## Data Availability

All data used to perform the application described in this paper are freely available. The data of van’t Veer et al. is available on the Gene Expression Omnibus data base [https://www.ncbi.nlm.nih.gov/geo/], series GSE2990. The data of Buyse et al. is available on the European Bioinformatics Institute ArrayExpress database [http://www.ebi.ac.uk/arrayexpress/], accession number E-TABM-77.

## Abbreviations

MM: Minorization-maximization
AUC: Area under curve
BID: Bayes information criteria
CLASSO: Cross least absolute shrinkage and selection operator
DS: Dependent setting
EM: Expectation–maximization
IS: Independent setting
MSE: Mean-squared error
