# Supplementary material for: Quasi-linear Cox proportional hazards model with cross- L1 penalty
Source: BMC Med Res Methodol. 2020 Jul 6;20:182. doi: 10.1186/s12874-020-01063-2 (PMC7336640; doi:10.1186/s12874-020-01063-2)
Supplement: Supplementary file 1 — Additional file 1 Technical Derivations. In this file, we give proofs of Proposition 1, Proposition 2, Theorem 1 and Theorem 2. [file 12874_2020_1063_MOESM1_ESM.pdf]

Appendix A : Proof of Proposition 1

Proof.

First, we note that the minus Hessian matrix of $G(\boldsymbol{\theta}, \boldsymbol{\theta}_0)$ with respect to $\boldsymbol{\theta}$ is positive-definite because

$$\frac{\partial^2}{\partial \pi_m \partial \pi_{m'}} G(\boldsymbol{\theta}, \boldsymbol{\theta}_0) = -\sum_{i=1}^{n} \delta_i I_{m',m} \frac{p_{m'i}(\boldsymbol{\theta}_0)}{\pi_{m'}^2},$$

$$\frac{\partial^2}{\partial \pi_m \partial \boldsymbol{\beta}_{m'}} G(\boldsymbol{\theta}, \boldsymbol{\theta}_0) = 0$$

and

$$\frac{\partial^2}{\partial \boldsymbol{\beta}_m \partial \boldsymbol{\beta}_{m'}} G(\boldsymbol{\theta}, \boldsymbol{\theta}_0) = -\sum_{i=1}^{n} I_{m',m}\ \delta_i \frac{\sum_{\ell \in R(t_i)} \pi_{m'} \exp(\boldsymbol{\beta}_{m'}^\top \boldsymbol{x}_\ell) \boldsymbol{x}_\ell \boldsymbol{x}_\ell^\top}{\sum_{\ell \in R(t_i)} \sum_{k=1}^{K} \pi_{0k} \exp(\boldsymbol{\beta}_{0k}^\top \boldsymbol{x}_\ell)},$$

where $I_{m,m'}$ is 1 if $m = m^{'}$ and 0 otherwise. This guarantees that $\boldsymbol{\theta}^{(s+1)} = (\tilde{\pi}_k(\boldsymbol{\theta}^{(s)}), \tilde{\boldsymbol{\beta}}_k(\boldsymbol{\theta}^{(s)}))$ is the unique minimizer of $G(\boldsymbol{\theta}, \boldsymbol{\theta}^{(s)})$ in $\boldsymbol{\theta}$. It follows that

$$\begin{aligned} \log \sum_{k=1}^{K} \pi_k \exp(\boldsymbol{\beta}_k^\top \boldsymbol{x}_i) \quad \geq \quad & \log \sum_{k=1}^{K} \pi_{0k} \exp(\boldsymbol{\beta}_{0k}^\top \boldsymbol{x}_i) \\ - \quad & \sum_{k=1}^{K} p_{ki}(\boldsymbol{\theta}_0) \log \frac{\pi_{0k} \exp(\boldsymbol{\beta}_{0k}^\top \boldsymbol{x}_i)}{\pi_k \exp(\boldsymbol{\beta}_k^\top \boldsymbol{x}_i)} \end{aligned} \tag{A.1}$$

because

$$\sum_{k=1}^{K} p_{ki}(\boldsymbol{\theta}_0) \log \frac{p_{ki}(\boldsymbol{\theta}_0)}{p_{ki}(\boldsymbol{\theta})} \geq 0.$$

Furthermore,

$$\begin{aligned} & -\log \sum_{\ell \in R(t_i)} \sum_{k=1}^{K} \pi_k \exp(\boldsymbol{\beta}_k^\top \boldsymbol{x}_\ell) \geq -\log \sum_{\ell \in R(t_i)} \sum_{k=1}^{K} \pi_{0k} \exp(\boldsymbol{\beta}_{0k}^\top \boldsymbol{x}_\ell) \\ & -\frac{\sum_{\ell \in R(t_i)} \sum_{k=1}^{K} \pi_k \exp(\boldsymbol{\beta}_k^\top \boldsymbol{x}_\ell)}{\sum_{\ell \in R(t_i)} \sum_{k=1}^{K} \pi_{0k} \exp(\boldsymbol{\beta}_{0k}^\top \boldsymbol{x}_\ell)} + 1 \end{aligned} \tag{A.2}$$

because it follows from the convexity of $-\log$ that

$$-\log A \geq -\log A_0 - \frac{A}{A_0} + 1.$$

In accordance with $(A.1)$ and $(A.2)$, we conclude that

$$l(\boldsymbol{\theta}) \geq G(\boldsymbol{\theta}, \boldsymbol{\theta}_0),$$

which implies that $l(\boldsymbol{\theta}^{(s+1)}) \geq G(\boldsymbol{\theta}^{(s+1)}, \boldsymbol{\theta}^{(s)})$ and

$$G(\boldsymbol{\theta}^{(s+1)}, \boldsymbol{\theta}^{(s)}) \geq G(\boldsymbol{\theta}^{(s)}, \boldsymbol{\theta}^{(s)}) = l(\boldsymbol{\theta}^{(s)})$$

from the fact that $G(\boldsymbol{\theta}^{(s+1)}, \boldsymbol{\theta}^{(s)}) \geq G(\boldsymbol{\theta}^{(s)}, \boldsymbol{\theta}^{(s)})$ and the definition of G. This results $l(\boldsymbol{\theta}^{(s+1)}) \geq l(\boldsymbol{\theta}^{(s)})$ for $s = 1, 2, \cdots, S-1$.

Appendix B : Proof of Proposition 2

Proof.

Let $\boldsymbol{\beta}$ and $\boldsymbol{\gamma}$ be in $\tilde{\mathcal{R}}_c$. Then, we observe that a vector $\boldsymbol{\beta}_q = q\boldsymbol{\beta} + (1-q)\boldsymbol{\gamma}$ is in $\tilde{\mathcal{R}}_c$ for $q, 0 < q < 1$. This is because

$$\begin{aligned}
\sum_{k\neq\ell}\sum_{j=1}^{p} |\beta_{q_{kj}}\beta_{q_{\ell j}}| &= \sum_{k\neq\ell}\sum_{j=1}^{p} |(q\beta_{kj} + (1-q)\gamma_{kj})(q\beta_{\ell j} + (1-q)\gamma_{\ell j})| \\
&= q^2 \sum_{k\neq\ell}\sum_{j=1}^{p} |\beta_{kj}\beta_{\ell j}| + (1-q)^2 \sum_{k\neq\ell}\sum_{j=1}^{p} |\gamma_{kj}\gamma_{\ell j}| + 2q(1-q) \sum_{k\neq\ell}\sum_{j=1}^{p} |\beta_{kj}\gamma_{\ell j}| \\
&\leq q^2 c + (1-q)^2 c + 2q(1-q) \sum_{k\neq\ell}\sum_{j=1}^{p} |\beta_{kj}\gamma_{\ell j}| \\
&\leq c
\end{aligned}$$

noting that

$$\sum_{k\neq\ell}\sum_{j=1}^{p} |\beta_{kj}\gamma_{\ell j}| \leq \sum_{k\neq\ell} \|\boldsymbol{\beta}_k\|_2 \|\boldsymbol{\gamma}_\ell\|_2 \leq \|\boldsymbol{\beta}\|_2 \|\boldsymbol{\gamma}\|_2 \leq c.$$

We conclude that $\tilde{\mathcal{R}}_c$ is a convex set for all $c \geq 0$.

Appendix C : Proof of Theorem 1

We introduce the following symbols and assume the regularity conditions as introduced in [1] and [2] noted in Appendix C. Suppose that individuals are under observation at times $t \in [0,1]$ and define some processes as $S^{(0)}(\boldsymbol{\theta}, t) = n^{-1}\sum_{\ell=1}^{n} Y_\ell(t) r(\boldsymbol{x_\ell}, \boldsymbol{\theta})$, $S^{(1)}(\boldsymbol{\theta}, t) = \partial S^{(0)}(\boldsymbol{\theta}, t)/\partial\boldsymbol{\theta}$, $S^{(2)}(\boldsymbol{\theta}, t) = n^{-1}\sum_{\ell=1}^{n} Y_\ell(t)\dot{r}(\boldsymbol{x_\ell}, \boldsymbol{\theta})^2/r(\boldsymbol{x_\ell}, \boldsymbol{\theta})$, $S^{(3)}(\boldsymbol{\theta}, t) = \partial^2 S^{(0)}(\boldsymbol{\theta}, t)/\partial\boldsymbol{\theta}\partial\boldsymbol{\theta}^\top$, $S^{(4)}(\boldsymbol{\theta}, t) = n^{-1}\sum_{\ell=1}^{n} Y_\ell(t)[u(\boldsymbol{x}, \boldsymbol{\theta}) - u(\boldsymbol{x}, \boldsymbol{\theta}_0)] r(\boldsymbol{x}, \boldsymbol{\theta}_0)$, $S^{(5)}(\boldsymbol{\theta}, t) = \partial S^{(4)}(\boldsymbol{\theta}, t)/\partial\boldsymbol{\theta}$, $S^{(6)}(\boldsymbol{\theta}, t) = \partial^2 S^{(4)}(\boldsymbol{\theta}, t)/\partial\boldsymbol{\theta}\partial\boldsymbol{\theta}^\top$, where $r(\boldsymbol{x}, \boldsymbol{\theta}) = \exp(f_Q(\boldsymbol{x}, \boldsymbol{\theta}))$ and $u(\boldsymbol{x}, \boldsymbol{\theta}) = \log(r(\boldsymbol{x}, \boldsymbol{\theta}))$. Here, $\dot{r}$ is the first derivative of function r. Assume the following regularity conditions.

Regularity Conditions:

A. $\int_0^1 h_0(t)dt < \infty$

B. There exists a neighborhood Θ of $\boldsymbol{\theta}_0$ and functions $s^{(j)}$ defined on $\Theta \times [0,1]$ such that

$$\sup_{t\in[0,1],\theta\in\Theta} \left\| S^{(j)}(\boldsymbol{\theta}, t) - s^{(j)}(\boldsymbol{\theta}, t) \right\| \xrightarrow{p} 0$$

for $j = 0, 1, \cdots, 6$, where $\|\cdot\|$ denotes the supremum norm.

C. Let $e = s^{(1)}/s^{(0)}$ and $v = s^{(2)}/s^{(0)} - ee^\top$. For each $j = 0, 1, \cdots, 6$, $s^{(j)}(\cdot, t)$ are continuous functions of $\boldsymbol{\theta} \in \Theta$, uniformly in $t \in [0,1]$. Also, $s^{(j)}, j = 0, 1, \cdots, 6$ are bounded on $\Theta \times [0,1]$, $s^{(0)}$ is bounded away from zero and the matrix

$$\Sigma = \int_0^1 v(\boldsymbol{\theta}_0, t) s^{(0)}(\boldsymbol{\theta}_0, t) h_0(t)dt$$

is positive definite. Also, $s^{(0)}(\boldsymbol{\theta}, t)$ is assumed to be twice differentiable with respect to $\boldsymbol{\theta}$ on $\Theta \times [0,1]$.

D.

$$\sup_{\boldsymbol{\theta}\in\Theta} \int_0^1 n^{-2} \sum_{i=1}^n Y_i(u) \|\boldsymbol{\theta} - \boldsymbol{\theta}_0\|^4 \ddot{u}(\boldsymbol{\theta})^2 h_0(u) du \xrightarrow{p} 0$$

Proof of Theorem 1 (1).

For $\boldsymbol{\theta} \in \Theta$ in assumption B, we define $X_n(\boldsymbol{\theta}, t) = n^{-1}[l(\boldsymbol{\theta}, t) - l(\boldsymbol{\theta}_0, t)]$. This is the difference in log partial likelihoods over $[0, t]$, evaluated at parameter $\boldsymbol{\theta}$ and the true value $\boldsymbol{\theta}_0$. Then it is written by the counting processes $N_i(t) = I(T_i \leq t, \delta_i = 1)$ and at risk processes $Y_i(t) = I(T_i \geq t)$ as

$$X_n(\boldsymbol{\theta}, t) \quad = \quad n^{-1} \int_0^t \left\{ \sum_{i=1}^n \log \frac{r(\boldsymbol{x}_i, \boldsymbol{\theta})}{r(\boldsymbol{x}_i, \boldsymbol{\theta}_0)} dN_i(u) - \log \frac{\sum_{\ell=1}^n Y_\ell(u) r(\boldsymbol{x}_\ell, \boldsymbol{\theta})}{\sum_{\ell=1}^n Y_\ell(u) r(\boldsymbol{x}_\ell, \boldsymbol{\theta}_0)} d\tilde{N}(u) \right\}$$

where $\tilde{N} = \sum_{i=1}^n N_i$. Let A_n be the corresponding value of X_n with replacement from integrator N_i to that compensator Λ_i. Then we get that

$$\begin{aligned} A_n(\boldsymbol{\theta}, t) \quad &= \quad n^{-1} \int_0^t \left\{ \sum_{i=1}^n \log \frac{r(\boldsymbol{x}_i, \boldsymbol{\theta})}{r(\boldsymbol{x}_i, \boldsymbol{\theta}_0)} d\Lambda_i(u) \right. \\ &- \quad \left. \log \frac{\sum_{\ell=1}^n Y_\ell(t) r(\boldsymbol{x}_\ell, \boldsymbol{\theta})}{\sum_{\ell=1}^n Y_\ell(t) r(\boldsymbol{x}_\ell, \boldsymbol{\theta}_0)} d\tilde{\Lambda}(u) \right\}, \end{aligned} \tag{A.3}$$

where $\tilde{\Lambda} = \sum_{i=1}^n \Lambda_i$. Let M be the martingale process of N. By the relationship $N = \Lambda + M$, we get that

$$\begin{aligned} X_n(\boldsymbol{\theta}, t) - A_n(\boldsymbol{\theta}, t) \quad &= \quad n^{-1} \int_0^t \left\{ \sum_{i=1}^n \log \frac{r(\boldsymbol{x}_i, \boldsymbol{\theta})}{r(\boldsymbol{x}_i, \boldsymbol{\theta}_0)} dM_i(u) - \log \frac{\sum_{\ell=1}^n Y_\ell(u) r(\boldsymbol{x}_\ell, \boldsymbol{\theta})}{\sum_{\ell=1}^n Y_\ell(u) r(\boldsymbol{x}_\ell, \boldsymbol{\theta}_0)} d\tilde{M}(u) \right\} \\ &= \quad \int_0^t \sum_{i=1}^n H_i dM_i, \end{aligned}$$

where $\tilde{M} = \sum_{i=1}^n M_i$ and $H_i = n^{-1} \log \frac{r(\boldsymbol{x}_i, \boldsymbol{\theta})}{r(\boldsymbol{x}_i, \boldsymbol{\theta}_0)} - n^{-1} \log \frac{\sum_{\ell=1}^n Y_\ell(u) r(\boldsymbol{x}_\ell, \boldsymbol{\theta})}{\sum_{\ell=1}^n Y_\ell(u) r(\boldsymbol{x}_\ell, \boldsymbol{\theta}_0)}$. We find that there exists $\epsilon > 0$ such that $r(\boldsymbol{x}_i, \boldsymbol{\theta}) > \epsilon$ for all $\boldsymbol{\theta} \in \Theta$ and so H_i is local bounded. Because Y_i is a left-continuous adapted process, Y_i is predictable, which implies that H_i is predictable. Therefore, H_i is a local-bounded $\mathcal{F}_t$-predictable process. Moreover, $M_i = N_i - \Lambda_i$ is a local square integrable martingale, by the Theorem 2.3.1 in [3]. Finally, we find that by the Theorem 2.4.5 in [3], $X_n(\boldsymbol{\theta}, t) - A_n(\boldsymbol{\theta}, t)$ is a local square integrable martingale. By Theorem 2.5.2 in [3], the variance process

$B(\boldsymbol{\theta}, t)$ is given by

$$\begin{aligned}
B(\boldsymbol{\theta}, t) &= \langle X_n - A_n, X_n - A_n \rangle \\
&= \left\langle \int_0^t \sum_{i=1}^n H_i dM_i, \int_0^t \sum_{i=1}^n H_i dM_i \right\rangle \\
&= \int_0^t \sum_{i=1}^n \sum_{j=1}^n H_i H_j d\langle M_i, M_j \rangle \\
&= \int_0^t \sum_{i=1}^n H_i^2 d\langle M_i, M_i \rangle \\
&= \int_0^t n^{-2} \sum_{i=1}^n \left[\log \frac{r(\boldsymbol{x}_i, \boldsymbol{\theta})}{r(\boldsymbol{x}_i, \boldsymbol{\theta}_0)} - \log \frac{\sum_{\ell=1}^n Y_\ell(u) r(\boldsymbol{x}_\ell, \boldsymbol{\theta})}{\sum_{\ell=1}^n Y_\ell(u) r(\boldsymbol{x}_\ell, \boldsymbol{\theta}_0)} \right]^2 \lambda_i(u) du.
\end{aligned}$$

Then we get that

$$\begin{aligned}
B(\boldsymbol{\theta}, 1) &= \int_0^1 n^{-2} \sum_{i=1}^n Y_i(u) \left[\log \frac{r(\boldsymbol{x}_i, \boldsymbol{\theta})}{r(\boldsymbol{x}_i, \boldsymbol{\theta}_0)} \right]^2 r(\boldsymbol{x}_i, \boldsymbol{\theta}) h_0(u) du \\
&- 2 \int_0^1 n^{-2} \sum_{i=1}^n Y_i(u) \log \frac{r(\boldsymbol{x}_i, \boldsymbol{\theta})}{r(\boldsymbol{x}_i, \boldsymbol{\theta}_0)} \cdot \log \frac{\sum_{\ell=1}^n Y_\ell(u) r(\boldsymbol{x}_\ell, \boldsymbol{\theta})}{\sum_{\ell=1}^n Y_\ell(u) r(\boldsymbol{x}_\ell, \boldsymbol{\theta}_0)} r(\boldsymbol{x}_i, \boldsymbol{\theta}) h_0(u) du \\
&+ \int_0^1 n^{-2} \left[\log \frac{\sum_{\ell=1}^n Y_\ell(u) r(\boldsymbol{x}_\ell, \boldsymbol{\theta})}{\sum_{\ell=1}^n Y_\ell(u) r(\boldsymbol{x}_\ell, \boldsymbol{\theta}_0)} \right]^2 \sum_{\ell=1}^n Y_\ell(u) r(\boldsymbol{x}_\ell, \boldsymbol{\theta}_0) h_0(u) du \qquad \text{(A.4)}
\end{aligned}$$

The final integral in $(A.4)$ converges in probability to zero by condition A, B and C on $S^{(0)}$. By the Schwarz inequality, the middle integral will converge in probability to zero if the first integral does so. The first integral also converges to zero, as follows. First, the Taylor expansion about θ_0 of $\log r(\boldsymbol{x}_i, \boldsymbol{\theta})/r(\boldsymbol{x}_i, \boldsymbol{\theta_0})$ is written as

$$\log \frac{r(\boldsymbol{x}_i, \boldsymbol{\theta})}{r(\boldsymbol{x}_i, \boldsymbol{\theta}_0)} = (\boldsymbol{\theta} - \boldsymbol{\theta}_0)^\top \dot{u}(\boldsymbol{\theta}_0) + \frac{1}{2} (\boldsymbol{\theta} - \boldsymbol{\theta}_0)^\top \ddot{u}(\boldsymbol{\theta}_*)(\boldsymbol{\theta} - \boldsymbol{\theta}_0),$$

where $\boldsymbol{\theta}^*$ is between $\boldsymbol{\theta}$ and $\boldsymbol{\theta}_0$. Then

$$\begin{aligned}
&\int_0^1 n^{-2} \sum_{i=1}^n Y_i(u) \left[\log \frac{r(\boldsymbol{x}_i, \boldsymbol{\theta})}{r(\boldsymbol{x}_i, \boldsymbol{\theta}_0)} \right]^2 r(\boldsymbol{x}_i, \boldsymbol{\theta}) h_0(u) du \\
= \; & (\boldsymbol{\theta} - \boldsymbol{\theta}_0)^\top \left[\int_0^1 n^{-2} \sum_{i=1}^n Y_i(u) \dot{u}(\boldsymbol{\theta}_0)^\top \dot{u}(\boldsymbol{\theta}_0) r(\boldsymbol{x}_i, \boldsymbol{\theta}) h_0(u) du \right] (\boldsymbol{\theta} - \boldsymbol{\theta}_0) \\
+ \; & (\boldsymbol{\theta} - \boldsymbol{\theta}_0)^\top \left[\int_0^1 n^{-2} \sum_{i=1}^n Y_i(u) \ddot{u}(\boldsymbol{\theta}_*)(\boldsymbol{\theta} - \boldsymbol{\theta}_0)(\boldsymbol{\theta} - \boldsymbol{\theta}_0)^\top r(\boldsymbol{x}_i, \boldsymbol{\theta}) h_0(u) du \right] (\boldsymbol{\theta} - \boldsymbol{\theta}_0) \\
+ \; & \frac{1}{4} (\boldsymbol{\theta} - \boldsymbol{\theta}_0)^\top \left[\int_0^1 n^{-2} \sum_{i=1}^n Y_i(u) \ddot{u}(\boldsymbol{\theta}_*)(\boldsymbol{\theta} - \boldsymbol{\theta}_0)(\boldsymbol{\theta} - \boldsymbol{\theta}_0)^\top \ddot{u}(\boldsymbol{\theta}_*) h_0(u) du \right] (\boldsymbol{\theta} - \boldsymbol{\theta}_0).
\end{aligned}$$

The first integral converges in probability to zero by condition A and B on $S^{(1)}$ in accordance with $S^{(5)} = S^{(1)}$. By the Schwarz inequality, the middle integral will converge in probability to zero if the final integral does so. For the final term it

follows that

$$\frac{1}{4}(\boldsymbol{\theta}-\boldsymbol{\theta}_0)^\top \left[\int_0^1 n^{-2}\sum_{i=1}^n Y_i(u)\ddot{u}(\theta_*)(\boldsymbol{\theta}-\boldsymbol{\theta}_0)(\boldsymbol{\theta}-\boldsymbol{\theta}_0)^\top \ddot{u}(\boldsymbol{\theta}_*)h_0(u)du\right](\boldsymbol{\theta}-\boldsymbol{\theta}_0)$$
$$\leq \frac{1}{4}\left[\int_0^1 n^{-2}\sum_{i=1}^n Y_i(u)\left\|\boldsymbol{\theta}-\boldsymbol{\theta}_0\right\|^4 \ddot{u}(\boldsymbol{\theta}_*)^2 h_0(u)du\right]$$

which converges in probability to zero by condition D. Therefore, $B(\boldsymbol{\theta},1)$ converges in probability to zero. By an inequality of Lenglart in [1], $X(\boldsymbol{\theta},1)$ converges in probability to the same limit with $A(\boldsymbol{\theta},1)$ for every $\boldsymbol{\theta}\in\Theta$. By conditions A and B, it follows that

$$A(\boldsymbol{\theta},1)\xrightarrow{p}\int_0^1\left\{s^{(4)}(\boldsymbol{\theta},u)-\log\frac{s^{(0)}(\boldsymbol{\theta},u)}{s^{(0)}(\boldsymbol{\theta}_0,u)}s^{(0)}(\boldsymbol{\theta}_0,u)\right\}h_0(u)du$$

As discussed in [1] and [2], we can take the first and second derivatives of the limit of $A(\boldsymbol{\theta},1)$ by taking partial derivatives inside the integral. This gives the first derivative

$$\int_0^1\left\{s^{(5)}(\boldsymbol{\theta},u)-\frac{s^{(1)}(\boldsymbol{\theta},u)}{s^{(0)}(\boldsymbol{\theta},u)}s^{(0)}(\boldsymbol{\theta}_0,u)\right\}h_0(u)du$$

which is equal to zero at $\boldsymbol{\theta}=\boldsymbol{\theta}_0$ and the second derivative

$$\int_0^1\left\{s^{(6)}(\boldsymbol{\theta},u)-\left\{\frac{s^{(3)}(\boldsymbol{\theta},u)}{s^{(0)}(\boldsymbol{\theta},u)}-\frac{s^{(1)}(\boldsymbol{\theta},u){s^{(1)}}^\top(\boldsymbol{\theta},u)}{s^{(0)}(\boldsymbol{\theta},u)}\right\}s^{(0)}(\boldsymbol{\theta}_0,u)\right\}h_0(u)du$$

which at $\boldsymbol{\theta}=\boldsymbol{\theta}_0$ equals $-\Sigma$, which by condition C is positive-definite matrix. Here, we note that $S^{(6)}(\boldsymbol{\theta}_0,t)=S^{(3)}(\boldsymbol{\theta}_0,t)-S^{(2)}(\boldsymbol{\theta}_0,t)$. It now follows that $X(\boldsymbol{\theta},1)$ converges in probability to a concave function h of $\boldsymbol{\theta}$ with a unique maximum at $\boldsymbol{\theta}=\boldsymbol{\theta}_0$. We find that $\hat{\boldsymbol{\theta}}\xrightarrow{p}\boldsymbol{\theta}_0$ by Appendix 2 in [1]. This completes the proof.

Proof of Theorem 1 (2).

Consider the score statistics defined by

$$U(\boldsymbol{\theta})=n^{-1/2}\sum_{i=1}^n\int_0^1\left\{\frac{\dot{r}(x_i,\boldsymbol{\theta})}{r(x_i,\boldsymbol{\theta})}-\frac{S^{(1)}(\boldsymbol{\theta},u)}{S^{(0)}(\boldsymbol{\theta},u)}\right\}dN_i(u)$$

and the Taylor series expansion for U centered at the true value $\boldsymbol{\theta}_0$ of $\boldsymbol{\theta}$ as

$$U(\hat{\boldsymbol{\theta}})=U(\boldsymbol{\theta}_0)-\mathcal{I}(\boldsymbol{\theta}^*)(\hat{\boldsymbol{\theta}}-\boldsymbol{\theta}_0),$$

where $\mathcal{I}(\boldsymbol{\theta}^*)$ is the observed information matrix at $\boldsymbol{\theta}^*$, which is on the line between $\boldsymbol{\theta}_0$ and $\hat{\boldsymbol{\theta}}$. Because $U(\hat{\boldsymbol{\theta}})=0$,

$$\left\{n^{-1}\mathcal{I}(\boldsymbol{\theta}^*)\right\}\left\{\sqrt{n}(\hat{\boldsymbol{\theta}}-\boldsymbol{\theta}_0)\right\}=n^{-1/2}U(\boldsymbol{\theta}_0).$$

By the Theorem 8.2.1 in [3], when conditions A-D hold, it follows that $n^{-1/2}U(\boldsymbol{\theta}_0)$ is asymptotically normal with mean zero and covariance Σ in condition D. By

the consistency of $\hat{\boldsymbol{\theta}}$, it follows that $\boldsymbol{\theta}^* \xrightarrow{p} \boldsymbol{\theta}_0$. Because $\mathcal{I}(\boldsymbol{\theta}^*) \xrightarrow{p} \Sigma$, we get that $\sqrt{n}(\hat{\boldsymbol{\theta}}-\boldsymbol{\theta}_0)$ has the same limiting distribution with $\sqrt{n}\mathcal{I}(\boldsymbol{\theta}^*)^{-1}U(\boldsymbol{\theta}_0) \xrightarrow{d} \mathrm{N}(\mathbf{0}, \Sigma^{-1})$, from the Slutsky's theorem. This completes the proof.

Appendix C : Proof of Theorem 2

Proof.

We assume the same regularity conditions noted in Appendix C. Let $l_n(\boldsymbol{\theta}) = l(\boldsymbol{\theta})$ with emphasizing the sample size n. In the same manner discussed in [4],

$$\frac{1}{n}\left\{l_n(\boldsymbol{\theta}_0 + n^{-1/2}\boldsymbol{v}) - l_n(\boldsymbol{\theta}_0)\right\} = -\frac{1}{2n}M^2 O_p(1)$$

for sufficiently large $||\boldsymbol{v}|| = M$, where $||\cdot||$ denotes L_2-norm. It is therefore sufficient to show that there exists a large constant $||\boldsymbol{v}|| = M$ such that

$$-\frac{1}{2n}M^2 O_p(1) + D_n(\boldsymbol{v}) \leq 0,$$

where $D_n(\boldsymbol{v}) = P_n(\boldsymbol{\beta}_0 + v_{\boldsymbol{\beta}}) - P_n(\boldsymbol{\beta}_0)$ and $P_n(\boldsymbol{\beta}) = n\lambda_c \sum_{\ell\neq m}\sum_{j=1}^{p} \frac{|\beta_{\ell j}\beta_{mj}|}{|\hat{\beta}_{\ell j}\hat{\beta}_{mj}|}$. We note that in the main paper we denotes $P_n(\boldsymbol{\beta})$ as $P_c(\boldsymbol{\beta})$ but exchange the subscript to emphasize the sample size. It follows that

$$\begin{aligned} D_n(\boldsymbol{v}) &= \lambda_n \sum_{\ell\neq m}\sum_{j=1}^{p} \frac{1}{|\hat{\beta}_{\ell j}\hat{\beta}_{mj}|}\left\{|(\beta_{0\ell j} + n^{-1/2}v_{\ell j})(\beta_{0mj} + n^{-1/2}v_{mj})| - |\beta_{0\ell j}\beta_{0mj}|\right\} \\ &\leq \lambda_n \sum_{\ell\neq m}\sum_{j=1}^{p} \frac{1}{|\hat{\beta}_{\ell j}\hat{\beta}_{mj}|}\left\{n^{-1/2}|\beta_{0\ell j}v_{mj} + \beta_{0mj}v_{\ell j}| + n^{-1}|v_{\ell j}v_{mj}|\right\} \\ &= \lambda_n \sum_{\ell\neq m}\sum_{j=1}^{p}\left\{n^{-1/2}\left(\left|\frac{\beta_{0\ell j}}{\hat{\beta}_{\ell j}}\right|\left|\frac{v_{mj}}{\hat{\beta}_{mj}}\right| + \left|\frac{\beta_{0mj}}{\hat{\beta}_{mj}}\right|\left|\frac{v_{\ell j}}{\hat{\beta}_{\ell j}}\right|\right) + n^{-1}\left|\frac{v_{\ell j}}{\hat{\beta}_{mj}}\right|\left|\frac{v_{mj}}{\hat{\beta}_{\ell j}}\right|\right\}. \end{aligned}$$

By the first order Taylor expansion,

$$\frac{1}{|\hat{\beta}_{\ell,j}|} = \frac{1}{|\beta_{0\ell,j}|} - \frac{\mathrm{sign}(\beta_{0\ell,j})}{\beta_{0\ell,j}^2}(\hat{\beta}_{\ell,j} - \beta_{0\ell,j}) + o_p(||\hat{\beta}_{\ell,j} - \beta_{0\ell,j}||),$$

and we then find from the property of the partial maximum likelihood estimator that $||\hat{\boldsymbol{\beta}}_\ell - \boldsymbol{\beta}_{0\ell}|| = O_p(n^{-1/2})$. This results in

$$\frac{1}{|\hat{\beta}_{\ell,j}|} = \frac{1}{|\beta_{0\ell,j}|} + O_p(n^{-1/2}).$$

We therefore get that

$$\begin{aligned} \left|\frac{\beta_{0\ell j}}{\hat{\beta}_{\ell j}}\right|\left|\frac{v_{mj}}{\hat{\beta}_{mj}}\right| &\leq \frac{|v_{mj}|}{|\beta_{0mj}|}\left|(1 + n^{-1/2}|\beta_{0\ell j}|O_p(1))\right|\left|(1 + n^{-1/2}|\beta_{0mj}|O_p(1))\right| \\ &\leq \frac{|v_{mj}|}{|\beta_{0mj}|}\left(1 + n^{-1/2}O_p(1)(|\beta_{0\ell j}| + |\beta_{0mj}|) + n^{-1}O_p(1)|\beta_{0\ell j}\beta_{0mj}|\right). \end{aligned}$$

for j such that $\beta_{0mj} \neq 0$. Let $E_n(z_1, z_2) = 1 + n^{-1/2}O_p(1)(|z_1| + |z_2|) + n^{-1}O_p(1)|z_1 z_2|$, then

$$D_n(\boldsymbol{v}) - \frac{1}{2n}M^2 O_p(1) \leq n^{-1/2}\lambda_n \sum_{\ell \neq m} \sum_{\{j:\beta_{0\ell j}\neq 0, \beta_{0mj}\neq 0\}} \left\{ \left(\frac{|v_{\ell j}|}{|\beta_{0\ell j}|} + \frac{|v_{mj}|}{|\beta_{0mj}|} \right) E_n(\beta_{0\ell j}, \beta_{0mj}) \right\}$$
$$+ n^{-1}\lambda_n \sum_{\ell \neq m} \sum_{\{j:\beta_{0\ell j}\neq 0, \beta_{0mj}\neq 0\}} \left\{ \frac{|v_{\ell j}||v_{mj}|}{|\beta_{0\ell j}||\beta_{0mj}|} E_n(\beta_{0\ell j}, \beta_{0mj}) \right\} - \frac{1}{2n}M^2 O_p(1)$$

By the assumption $n^{-1/2}\lambda_n = O_p(1)$, for sufficient $||v|| = M$, $D_n(\boldsymbol{v}) \leq 0$. This completes the proof.

Author details

References

1. Andersen, P.K., Gill, R.D.: Cox's regression model for counting process: A large sample study. Annals of Statistics **10**, 1100–1120 (1982)
2. Prentice, R.L., Self, S.G.: Asymptotic distribution theory for cox-type regression models with general relative risk form. The Annals of Statistics **11**(3), 804–813 (1983)
3. Fleming, T.R., Harrington, D.P.: Counting Processes and Survival Analysis. New York: Wiley, ??? (1991)
4. You, N., He, S., Wang, X., Zhu, J., Zhang, H.: Subtype classification and heterogeneous prognosis model construction in precision medicine. Biometrics, 10–111112843 (2018)
